# Correlation between Progetto Cuore risk score and early cardiovascular damage in never treated subjects

**DOI:** 10.1186/1476-7120-6-47

**Published:** 2008-09-22

**Authors:** Aldo Pende, Caterina Grondona, Stefano Bertolini

**Affiliations:** 1 Department of Internal Medicine, University of Genoa School of Medicine, Viale Benedetto XV, 6, 16132 Genoa, Italy

## Abstract

**Background:**

Global cardiovascular risk is a new approach which allows the physicians to quantitate the prognosis of the patients. It is therefore possible that a score, based on the major cardiovascular risk factors, is correlated with some degree of cardiovascular anatomic damage. Since this hypothesis has been demonstrated with the Framingham risk score, we decided to verify it using another score (Progetto Cuore risk score), which is probably more precise in a european low-risk population, such as the italian one.

**Methods:**

We studied 84 italian caucasian subjects (50 males and 34 females) with elevated blood pressure and/or dyslipidemia plus other possible cardiovascular risk factors.

The subjects have never been treated for these reasons. The following evaluations were performed: history, clinical and laboratory determinations, echocardiogram, carotid echodoppler.

**Results:**

The recruited people were on the whole characterized by a low cardiovascular risk, as confirmed by the low scores of the Progetto Cuore. Simple linear regression analysis showed significant associations between some parameters of early cardiovascular damage (left ventricular mass, intima-media thickness, and an integrated measure of both the carotid wall thickness and the presence of a plaque, called Carotid score) and some predictors. The highest significance was found between the cardiovascular structural results and the Progetto Cuore score. In a multivariate regression analysis our model, which included factors potentially linked to the cardiovascular anatomic changes, demonstrated that the Carotid score was significantly associated with age, sex and pulse pressure; intima-media thickness with the same factors and, in addition, with the body mass index; left ventricular mass with sex, pulse pressure and body mass index.

**Conclusion:**

Our paper confirms previous studies about the association between a comprehensive risk score and signs of early cardiovascular damage. A temporally limited exposure to cardiovascular risk factors, in particular to blood pressure, is already able to induce significant changes in both the heart structure and the vascular wall. Also in a european low-risk population the use of a cardiovascular risk score program, such as the Progetto Cuore in Italy, allows a quite precise estimation of the possible cardiovascular damage.

## Background

The concept of global cardiovascular risk has been widely accepted in medicine: the patient is no more considered as a carrier of single cardiovascular risk factors, to be tackled one by one by the physician, but as a subject potentially affected by their interactions. This approach was proposed in 2003 by the guidelines of the European Society of Hypertension and the European Society of Cardiology [1], in partial disagreement with those written in the same year by the american hypertension specialists, which put the blood pressure values only as reference for the beginning of the anti-hypertensive therapy [2].

In support of this new approach in the last few years many projects were developed (in Italy named Progetto Cuore) in order to provide the practising physician a quantification of the cardiovascular risk of the single patient through a computerized procedure based on the acquisition of clinical parameters, such as age, sex, blood pressure values, lipid status, and smoking habits.

In order to evaluate the impact of different risk factors on target organs and the possible correlations with the italian risk score Progetto Cuore, we decided to study subjects sent to our outpatient clinic by the general practitioners. Our outpatient clinic is devoted to patients with dyslipidemia and hypertension: we could therefore choose to evaluate subjects with two important cardiovascular risk factors, such as high blood pressure and/or abnormal lipid values, but without previous or concomitant therapies.

## Methods

Since February 2007 through September 2007 we studied 84 caucasian subjects (50 males and 34 females) affected by blood pressure above normal levels (high normal blood pressure or hypertension) and/or dyslipidemia, sent to our outpatient clinic by the general practitioners in a local survey of the prevalence of the metabolic syndrome.

The inclusion criteria are shown in Table 1. In all subjects we obtained a history, a complete clinical examination, fasting blood and urine samples, and we performed a B-mode echocardiogram and a carotid echodoppler. The clinical parameters obtained with this approach are shown in Table 2. Blood pressure and heart rate are expressed as means of three values measured consecutively by a mercury sphygmomanometer and at the radial artery, respectively.

**Table 1:** Inclusion criteria

Age between 35 and 75 years
Presence of blood pressure values higher than 130/85 mmHg and/or dyslipidemia (defined according to the ESH/ESC guidelines)
Absence of previous or concomitant drug treatments
Absence of secondary hypertension and monogenic dyslipidemia
Absence of serious pulmonary, renal, hepatic, gastrointestinal, endocrine, immunologic, neoplastic diseases
Absence of previous cardiovascular diseases
Absence of diabetes mellitus
Absence of pregnancy

**Table 2:** Clinical characteristics of the subjects

**Number**	84
**Sex (Males/Females)**	50/34 (59/41%)
**Hypertension (Yes/No)**	48/36 (57/43%)
**Grades of Hypertension:**	
**1**	35 (73%)
**2**	12 (25%)
**3**	1 (2%)
**Types of Dyslipidemia:**	
**Total Cholesterol > 190**	73 (87%)
**HDL Cholesterol < 40**	19 (23%)
**Triglycerides > 150**	62 (74%)
**Smoke (Yes/No)**	24/60 (29/71%)
**Metabolic Syndrome (Yes/No)**	36/48 (43/57%)

Global cardiovascular risk at 10 years was evaluated using the Progetto Cuore program of the Istituto Superiore di Sanità : this score is calculated taking into account some variables such as sex, age, smoking habits, systolic and diastolic blood pressure, total cholesterol levels, HDL-cholesterol levels, and possible anti-hypertensive therapy.

The metabolic syndrome was defined using the criteria of the National Cholesterol Education Program – Adult Treatment Panel III (NCEP-ATP III) [3].

All the echocardiogram examinations were performed by the same experienced cardiologist using an ATL model HDI 5000 B-mode echocardiograph with a 3.5 MHz sectorial probe. Registrations were performed along the long axis parasternal projection with the M-mode measurement of the left atrium, aortic root, and left ventricle (end diastolic and end systolic thickness of both the ventricular septum and the posterior wall, end diastolic and end systolic diameters of the left ventricle). The doppler registration of the transmitralic profile was performed in the apical projection. Left ventricle mass (LVM) was determined calculating the left ventricle mass (according to the formula of the American Society of Echocardiography) [4]. The relative wall thickness of the left ventricle was calculated as the ratio between the double of the end diastolic posterior wall and the chamber diameter.

All the carotid echodopplers were performed by the same physician using ESAOTE model AU5 with a 7.5 MHz linear probe. The examination of the subjects, in supine position and with the extrarotated head contralaterally by 45°C, evaluated the common carotid artery, the carotid bifurcation and the extracranial tracts of both the internal and external carotids with a longitudinal and transverse approach. Intima-media thickness was determined in telediastole at the level of the far wall of the common carotid artery at a distance of 20-10-5 mm from the bifurcation. For statistical analysis the mean of the three measurements in both common carotid arteries (defined as mean intima-media thickness, m-IMT) was used. According to the ESH/ESC guidelines an intima-media thickness lower than 0.9 mm was considered normal; a thickness higher than 0.9 mm but lower than 1.3 was considered increased; a thickness higher than 1.3 mm was defined as a plaque [1]. Based on these definitions, we calculated a Carotid score giving 1 point (presence of plaque), 0.5 point (presence of increased intima-media thickness) and 0 points (absence of lesions): the final score was the sum of the possible lesions in 10 sites (3 at common carotid artery and at the bifurcation, 1 at internal carotid artery, and 1 at external carotid artery on both sides).

Statistical analysis was carried out using Statistica 8.0 package (StatSoft Inc., Tulsa, OK, USA). For statistical purposes the Progetto Cuore scores were log-transformed to reduce skewness.

## Results

The clinical characteristics of the subjects studied are shown in Tables 2 and 3. As we already mentioned, we evaluated subjects without previous treatments for hypertension and dyslipidemia. Since the inclusion criteria allowed the recruitment of subjects with mild increases of blood pressure and lipid values, our population was on the whole characterized by not advanced age, high normal mean blood pressure values, and not very high mean serum levels of total cholesterol and triglycerides.

**Table 3:** Parameters of the subjects

	**Mean**	**SD**
**Age (years)**	52.5	10.05
**Weight (kg)**	77.53	16.64
**Height (cm)**	169.67	9.94
**Body Mass Index (kg/m^2^)**	26.78	4.41
**Waist Circumference (cm)**	96.47	13.51
**Systolic Blood Pressure (mmHg)**	137.34	14.79
**Diastolic Blood Pressure (mmHg)**	88.2	9.43
**Pulse Pressure (mmHg)**	49.14	10.26
**Heart Rate (bpm)**	74.32	8.84
**Total Cholesterol (mg/dl)**	246.76	49.7
**HDL Cholesterol (mg/dl)**	52.99	15.7
**Total/HDL Cholesterol Ratio**	4.94	1.37
**Triglycerides (mg/dl)**	202.38	85
**Glucose (mg/dl)**	86.83	13.39
**Insulin (μU/ml)**	9.86	9.12
**Creatinine (mg/dl)**	0.85	0.16
**Uric Acid (mg/dl)**	5.44	1.48
**Components of the Metabolic Syndrome**	2.29	0.99
**Urinary Albumin/Creatinine Ratio (mg/g)**	13.92	16.61
**Left Ventricle Mass (g)**	146.08	40.9
**Relative Wall Thickness**	0.34	0.08
**m-IMT (mm)**	0.81	0.22
**Carotid Score (A.U.)**	2.02	1.77
**Progetto Cuore Score**	5.16	5.3

These characteristics were confirmed by the cardiovascular risk scores calculated for our subjects: mean value was 5.16 ± 5.3 SD, and only 2 of them (2%) were above 20% risk of cardiovascular events at 10 years (i.e. high cardiovascular risk according to the Progetto Cuore score).

**Figure 1:**
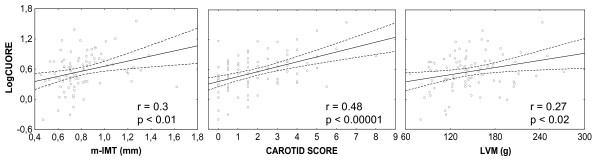
Univariate correlations between the logarithmic transformation of the Progetto Cuore score (LogCUORE) and the mean intima-media thickness (m-IMT), the Carotid score, and the left ventricular mass (LVM) in all subjects (n. = 84).

Simple linear regression analysis showed significant associations between some responses (Carotid score, m-IMT, LVM) and some predictors (Table 4 and Figure 1). Instead urinary albumin/creatinine ratio and relative wall thickness demonstrated no significant associations with any independent variable (data not shown). When we evaluated the possible associations between an integrated measurement such as the Progetto Cuore score and the previous dependent variables we found a very high significance with the Carotid score, the m-IMT, and the LVM.

**Table 4:** Simple regression analysis – All subjects. Only significant associations are shown.

**Variable**	**Carotid Score**	**m-IMT**	**Left Ventricular Mass**
**Age**	r = 0.41 p < 0.001	r = 0.23 p < 0.05	-
**Weight**	-	r = 0.23 p < 0.05	r = 0.75 p < 0.00001
**Height**	-	-	r = 0.53 p < 0.0001
**Body Mass Index**	-	-	r = 0.58 p < 00001
**Waist Circumference**	-	r = 0.22 p < 0.05	r = 0.66 p < 0.00001
**Systolic BP**	r = 0.26 p < 0.05	r = 0.27 p < 0.01	r = 0.29 r < 0.01
**Diastolic BP**	-	-	r = 0.26 p < 0.02
**Pulse Pressure**	r = 0.28 p < 0.05	r = 0.3 p < 0.01	-
**Heart Rate**	-	-	-
**Total Cholesterol**	-	-	r = -0.28 p < 0.01
**HDL Cholesterol**	-	-	r = -030 p < 0.01
**Total/HDL Ratio**	-	-	-
**Triglycerides**	-	-	-
**Glucose**	-	-	-
**Insulin**	-	-	r = 0.34 p < 0.01
**Creatinine**	-	-	r = 0.24 p < 0.05
**Uric acid**	-	-	r = 0.46 p < 0.0001
**Progetto Cuore** **Score**	r = 0.46 p < 0.001	r = 0.27 p < 0.02	r = 0.28 p < 0.01
**Progetto Cuore** **Score – Logarithm**	r = 0.48 p < 0.00001	r = 0.3 p < 0.01	r = 0.27 p < 0.02

We also divided the subjects according to the sex: in both male (Table 5) and female (Table 6) subjects we confirmed some significant associations but again the highest one was between the Carotid score and the Progetto Cuore score.

**Table 5:** Simple regression analysis – Male subjects (n = 50). Only significant associations are shown.

**Variable**	**Carotid Score**	**m-IMT**	**Left Ventricular Mass**
**Age**	r = 0.49 p < 0.001	-	-
**Weight**	-	-	r = 0.70 p < 0.00001
**Height**	-	-	-
**Body Mass Index**	-	-	r = 0.65 p < 00001
**Waist Circumference**	-	-	r = 0.64 p < 0.00001
**Systolic BP**	r = 0.29 p < 0.05	r = 0.33 p < 0.05	r = 0.32 r < 0.05
**Diastolic BP**	-	-	-
**Pulse Pressure**	r = 0.38 p < 0.01	r = 0.34 p < 0.05	-
**Heart Rate**	-	-	-
**Total Cholesterol**	-	-	-
**HDL Cholesterol**	-	-	-
**Total/HDL Ratio**	-	-	-
**Triglycerides**	-	-	-
**Glucose**	-	-	-
**Insulin**	-	-	r = 0.31 p < 0.05
**Creatinine**	-	-	-
**Uric acid**	-	-	r = 0.30 p < 0.05
**Progetto Cuore** **Score**	r = 0.46 p < 0.001	-	-
**Progetto Cuore** **Score – Logarithm**	r = 0.44 p < 0.01	-	-

**Table 6:** Simple regression analysis – Female subjects (n = 34). Only significant associations are shown.

**Variable**	**Carotid Score**	**m-IMT**	**Left Ventricular Mass**
**Age**	r = 0.53 p < 0.01	r = 0.46 p < 0.01	-
**Weight**	-	-	r = 0.53 p < 0.01
**Height**	-	-	r = 0.38 p < 0.05
**Body Mass Index**	-	-	-
**Waist Circumference**	-	-	r = 0.57 p < 0.001
**Systolic BP**	-	-	-
**Diastolic BP**	-	-	-
**Pulse Pressure**	-	-	-
**Heart Rate**	-	-	-
**Total Cholesterol**	-	-	-
**HDL Cholesterol**	-	-	-
**Total/HDL Ratio**	-	-	-
**Triglycerides**	-	-	-
**Glucose**	-	-	-
**Insulin**	-	-	-
**Creatinine**	-	-	-
**Uric acid**	-	-	-
**Progetto Cuore** **Score**	r = 0.55 p < 0.001	r = 0.36 p < 0.05	-
**Progetto Cuore** **Score – Logarithm**	r = 0.54 p < 0.01	r = 0.43 p < 0.05	-

In a multivariate regression analysis (Table 7), we used a model which included factors potentially linked to the Carotid score, to the m-IMT, to the left ventricle mass, and to the urinary albumin/creatinine ratio. For the Carotid score age, sex and pulse pressure maintained a significant association; for the m-IMT there was a low significance also with body mass index. On the contrary sex, pulse pressure and body mass index showed a significant association with the LVM.

**Table 7:** Multiple regression analysis. Only significant associations are shown.

**Variable**	**Carotid Score**	**m-IMT**	**Left Ventricular Mass**
**Age**	β = 0.542 p < 0.00001	β = 0.336 p < 0.01	-
**Sex**	β = 0.301 p < 0.01	β = 0.236 p < 0.05	β = 0.406 p < 0.0001
**Pulse Pressure**	β = 0.191 p < 0.05	β = 0.235 p < 0.05	β = 0.166 p < 0.05
**Tot/HDL Ratio**	-	-	-
**Glucose**	-	-	-
**Body Mass Index**	-	β = 0.274 p < 0.05	β = 0.571 p < 0.0001
**Smoke**	-	-	-

In addition m-IMT was significantly associated with LVM when we considered all the subjects; this association was confirmed in female subjects, instead it was not seen in male subjects (Table 8).

**Table 8:** Relation between vascular (m-IMT and Carotid score) and cardiac parameters (LVM).

	**Overall**	**Male**	**Female**
**m-IMT – LVM**	0.31 p < 0.01	n.s.	0.38 p < 0.05
**Carotid Score – LVM**	n.s.	n.s.	n.s.

As a final point, the presence of the metabolic syndrome was not able to induce a higher level of damage at the cardiovascular sites (data not shown).

## Discussion

Our results show that, in a population with some cardiovascular risk factors but, as a whole, not at particularly high risk, it is possible to demonstrate a significant correlation between a simple risk score, used in clinical practice by italian physicians, and some parameters of target organ damage. This was something already demonstrated with the Framingham risk score and confirmed by recent papers [5-7]. However it is well known that this cardiovascular score does not fit perfectly to our population with a tendency to the overestimation of the risk [8,9]: this is the main reason we decided to evaluate the correlation between target organ damage and the Progetto Cuore risk score.

In addition, the absence of the effects of any anti-hypertensive and hypolipemic therapy allows us to exclude possible drug effects in terms of reversal of the anatomic damage: this is something the other studies, although with more subjects, had to take into account.

Our population can be considered at not very long exposure of traditional risk factors: this can be inferred by the absence of drug therapy for hypertension or dyslipidemia. In fact all the subjects studied in this study were regularly evaluated by their family physicians and, at the appearance of an increase of blood pressure or blood lipids, rapidly sent to our clinic. In addition the age and the absolute levels of those parameters put our subjects on average at low risk of cardiovascular events; again in other studies all the categories of risk were included.

Our clinical evaluations allow to find interesting associations. The most significant is the association between Progetto Cuore score and the Carotid score: the associations with m-IMT and LVM were less significant. The reason we used the Carotid score in order to define the damage of the vascular wall was related to the possible different meanings of the intima-media thickness and the plaques [10,11]: in fact the increase of the intima-media thickness could represent a hypertrophic response of intimal and medial cells to vascular risk factors (hypertension, dyslipidemia); instead the atherosclerotic plaques could represent a later stage of vascular damage related to endothelial dysfunction and inflammation. A possible confirmation of this hypothesis is the presence of a significant relationship between m-IMT and LVM (another parameter mainly affected by the blood pressure levels), but not between LVM and Carotid score: previous studies showed that IMT was more closely associated with LVM than with coronary artery disease [12,13]. The use of a score for the evaluation of the carotid walls was proposed by some groups [14,15]; others suggested the determination of the plaque area [16].

When we performed the simple regression analysis, as a first step we evaluated the all subjects finding the significant associations shown in Table 4. Subsequently we divided the subjects by sex and we could not confirm all the statistical significances: however Progetto Cuore score maintained a significant association with the Carotid score in both sexes.

The multiple regression analysis was carried out defining a model with possible variables potentially related to both vascular and cardiac damage: for Carotid score we found significant associations with age, sex and pulse pressure. As for m-IMT, body mass index was significant in addition to the above mentioned variables. Instead with regards to the LVM the significant associations in the multiple regression analysis were with sex, pulse pressure and body mass index. These statistical evaluations suggest that, among modifiable cardiovascular risk factors, blood pressure is more important than cholesterol in inducing initial damage of heart and vessels.

An apparent contradiction in our results is a better correlation with the Progetto Cuore score exhibited by the carotid damage with respect to the heart: it is well known that the left ventricular hypertrophy is a better prognostic indicator in terms of cardiovascular events. Again we believe that our particular population (subjects with not a long exposure to risk factors) can explain this discrepancy. Only one subject could be considered with left ventricular hypertrophy (according to the ESH-ESC criteria) and in this setting of subjects a thorough vascular evaluation can be more sensitive than the echocardiographic examination.

A final issue of these results is the presence of rather low correlation coefficients, although with good statistical significance. This is something already shown in similar studies [7], likely related to the fact that the cardiovascular damage can be a marker of risk *per se*, indipendently of the usual risk factors [17,18].

## Conclusion

Our paper confirms previous studies about the association between a comprehensive risk score and signs of early cardiovascular damage. A temporally limited exposure to cardiovascular risk factors is already able to induce significant changes in both the heart structure and the vascular wall. Among the possible risk factors for these changes blood pressure seems to be the most powerful inducer.

Echographic examination of the cardiovascular system represents a useful tool for a complete prognostic evaluation of subjects with different risk factors.

## Competing interests

The authors declare that they have no competing interests.

## Authors' contributions

AP conceived the study, carried out the clinical evaluations, and drafted the manuscript. CG carried out the clinical evaluations and the ultrasound examinations. SB participated in the design of the study and helped to draft the manuscript. All the authors read and approved the final manuscript.
